# New Synthetic Lethality Re-Sensitizing Platinum-Refractory Cancer Cells to Cisplatin In Vitro: The Rationale to Co-Use PARP and ATM Inhibitors

**DOI:** 10.3390/ijms222413324

**Published:** 2021-12-11

**Authors:** Watson P. Folk, Alpana Kumari, Tetsushi Iwasaki, Erica K. Cassimere, Slovénie Pyndiah, Elizabeth Martin, Kelly Homlar, Daitoku Sakamuro

**Affiliations:** 1 Department of Biochemistry and Molecular Biology, Medical College of Georgia, Augusta University, Augusta, GA 30912, USA; watson.p.folk@gmail.com (W.P.F.); alpana01@gmail.com (A.K.); tiwasaki@kobe-u.ac.jp (T.I.); 2 Georgia Cancer Center, Augusta University, Augusta, GA 30912, USA; EMARTIN6@augusta.edu (E.M.); KHOMLAR@augusta.edu (K.H.); 3 Division of Signal Pathways, Biosignal Research Center, Kobe University, Kobe 657, Japan; 4 Department of Biology, College of Science, Engineering and Technology, Texas Southern University, Houston, TX 77004, USA; Erica.Cassimere@tsu.edu; 5 Les Francs Bourgeois-La Salle, 75004 Paris, France; slovenie.pyndiah@gmail.com; 6 Department of Pathology, Medical College of Georgia, Augusta University Medical Center, Augusta, GA 30912, USA; 7 Department of Orthopaedic Surgery, Medical College of Georgia, Augusta University Medical Center, Augusta, GA 30912, USA

**Keywords:** MYC, PARP1, ATM, BIN1, MDC1-RNF8 complex, apoptosis, cisplatin resistance

## Abstract

The pro-apoptotic tumor suppressor BIN1 inhibits the activities of the neoplastic transcription factor MYC, poly (ADP-ribose) polymerase-1 (PARP1), and ATM Ser/Thr kinase (ATM) by separate mechanisms. Although BIN1 deficits increase cancer-cell resistance to DNA-damaging chemotherapeutics, such as cisplatin, it is not fully understood when BIN1 deficiency occurs and how it provokes cisplatin resistance. Here, we report that the coordinated actions of MYC, PARP1, and ATM assist cancer cells in acquiring cisplatin resistance by *BIN1* deficits. Forced *BIN1* depletion compromised cisplatin sensitivity irrespective of Ser15-phosphorylated, pro-apoptotic TP53 tumor suppressor. The BIN1 deficit facilitated ATM to phosphorylate the DNA-damage-response (DDR) effectors, including MDC1. Consequently, another DDR protein, RNF8, bound to ATM-phosphorylated MDC1 and protected MDC1 from caspase-3-dependent proteolytic cleavage to hinder cisplatin sensitivity. Of note, long-term and repeated exposure to cisplatin naturally recapitulated the *BIN1* loss and accompanying RNF8-dependent cisplatin resistance. Simultaneously, endogenous MYC was remarkably activated by PARP1, thereby repressing the *BIN1* promoter, whereas PARP inhibition abolished the hyperactivated MYC-dependent *BIN1* suppression and restored cisplatin sensitivity. Since the *BIN1* gene rarely mutates in human cancers, our results suggest that simultaneous inhibition of PARP1 and ATM provokes a new BRCAness-independent synthetic lethal effect and ultimately re-establishes cisplatin sensitivity even in platinum-refractory cancer cells.

## 1. Introduction

Rosenberg et al. discovered the potent anti-cell division property of *cis-diammine dichloro platinum* (cisplatin or CDDP) first in *Escherichia coli* and then in tumor cells in the 1960s [1,2]. Inspired by this epoch-making finding, Einhorn et al. reported a remarkable therapeutic efficacy of cisplatin in patients with refractory testicular cancer in the 1970s [3,4]. Cisplatin has since been widely recognized as the first-line chemotherapy in various human malignancies [5,6]. Cisplatin frequently forms platinum-DNA adducts to interfere with DNA replication, transcription, and repair. As in thymine-dimer caused by ultraviolet conditions, platinum-DNA adducts are usually removed by the nucleotide excision repair (NER) pathway [7,8]. Unrepaired platinum-DNA adducts generate single-stranded DNA breaks (SSBs) during DNA replication, which are converted easily to double-stranded DNA breaks (DSBs), the most devastating form of DNA lesion, after the collapse of stalled replication forks [7,8]. The robust increase in DSBs could be the most likely cause of cisplatin-induced catastrophic cell death in cancer. Nonetheless, cisplatin-treated cancer cells eventually demonstrate the capability to survive even in the presence of cisplatin [5,6,7,8]. Because most cancer cells are initially susceptible to cisplatin, we assume that a cell death-promoting mechanism caused by DSBs could be impaired in advanced cancer cells. 

The bridging integrator 1 (BIN1) adaptor protein was identified initially as a pro-apoptotic tumor suppressor that binds to and suppresses the oncogenic MYC transcription factor [9,10,11,12,13]. *BIN1* is ubiquitously expressed in terminally differentiated normal tissues in various organs, but the biological purposes of BIN1 in normal growth-arrested tissues, where little or no MYC is detectable, are unclear [9,14,15]. BIN1 is often undetectable in refractory or metastatic cancer cells [16,17]. As with the TP53 tumor suppressor, BIN1 activates cell death due to DNA damage [18,19]. Conversely, BIN1 deficiency often accompanies cancer chemoresistance [19]. Therefore, it is reasonable to speculate that a BIN1 loss somehow activates cellular mechanisms vital for cancer-cell survival in the presence of genotoxic therapeutics, such as cisplatin. Consistent with this expectation, BIN1 directly inhibits the enzymatic activity of poly(ADP-ribose) polymerase-1 (PARP1) [19,20,21] and indirectly attenuates auto-phosphorylation of ataxia telangiectasia-mutated Ser/Thr kinase (ATM) [20,21]. Because PARP1 and ATM are essential for the maintenance of genome stability, it is crucial to identify the effectors of these enzymes provoked by a BIN1 loss to initiate and promote cisplatin resistance.

## 2. Results

### 2.1. BIN1 Broadly Retains Cancer-Cell Susceptibility to Cisplatin Regardless of TP53 Statuses

**Figure 1:**
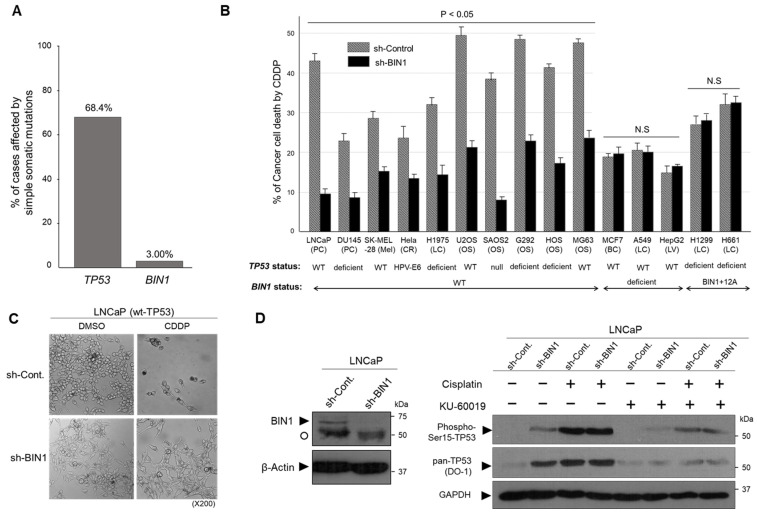
Forced BIN1 depletion widely decreases cisplatin sensitivity in various human cancer cell lines regardless of the *TP53* gene status. (**A**) The mutation ratio of the *TP53* and *BIN1* genes in human solid cancer tissues is available at the US NCI Genomic DNA Commons (https://gdc.cancer.gov [accessed on 1 June 2021]). Simple somatic mutations include missense mutations, frameshift mutations, start-loss mutations, stop-loss mutations, and stop-gained mutations in multiple human cancer tissues derived from various organs, such as the lung, breast, bone marrow, colon, nervous system, ovary, kidney, skin, and prostate; (**B**) the cisplatin-induced cell death percentage (%) of cultured cancer cell lines ± sh-BIN1. The growing cancer cells were incubated with cisplatin at 2.0 µg/mL for 72 h post-transfection, during which the cells were expected to complete at least one DNA replication cycle. The floating and adherent cell fractions were combined and subjected to the trypan-blue exclusion assay. PC: prostate cancer, Mel: melanoma, CR: cervical cancer, LC: lung cancer, OS: osteosarcoma, BC: breast cancer, LV: liver cancer, HPV-E6: human papillomavirus type-18 E6 protein, NS: not (statistically) significant; (**C**) the cellular morphology of LNCaP ± sh-BIN1 cells cultured in the presence of cisplatin (CDDP) (2.0 µg/mL for 72 h) or 1/100 (*v/v*) of DMSO (dimethyl sulfoxide) was monitored under phase-contrast microscopy; (**D**) Western blotting analysis of Ser15-phosphorylated TP53 and pan TP53 proteins in LNCaP ± sh-BIN1 cell lysates in the presence (+) and absence (−) of 2.0 µg/mL of cisplatin and/or 3.0 µM of KU-60019, an ATM-specific small molecule inhibitor at 24 h post-treatment [25]. β-Actin and GAPDH (glyceraldehyde 3-phosphate dehydrogenase) were used as the internal loading control. An open circle indicates a non-specific protein signal.

As with the pro-apoptotic tumor suppressor TP53, BIN1 increases cell death due to DNA damage [18,19,20,21]. Because the *TP53* gene is frequently mutated and inactivated in human cancers [22,23], we investigated how frequently the *BIN1* gene is mutated in the same sets of human cancers. In the U.S. National Cancer Institute (NCI) Genomic DNA Commons (https://gdc.cancer.gov/ [accessed on 1 June 2021]), where mutation incidences of various cancer-related genes are constantly updated, the present somatic mutations in the cohort analysis of the *TP53* gene detected in various human adult cancer tissues was 68.4% (4401 mutations in 6436 cases). In contrast, the corresponding mutation rate of the *BIN1* gene was merely 3.0% (193/6436) (Figure 1A). This data suggests that, unless BIN1-induced cancer-cell death depends on TP53, pharmacological activation of a less mutated cell death-promoting gene, such as *BIN1*, would be a more realistic strategy to eradicate cisplatin-resistant cancer cells than activating the *TP53* gene that might be already dysfunctional due to a gene mutation.

In wt-*BIN1*-expressing cancer cells, forced depletion of BIN1 by the transient transfection of the short-hairpin RNA directed against human *BIN1* mRNA (sh-BIN1) consistently compromised the cisplatin sensitivity irrespective of the expression status of *TP53*. In contrast, as predicted, the sh-*BIN1* transfection did not alter the cisplatin sensitivity in BIN1-deficient cancer cell lines. Interestingly, the cisplatin sensitivity of cancer cell lines expressing BIN1+12A, an aberrant alternative splicing variant of *BIN1* [24], was unchanged by sh-BIN1 (Figure 1B), suggesting that the impairment of wt-BIN1 by a BIN1 loss or aberrant alternative splicing abolishes the cisplatin sensitivity regardless of the *TP53* gene expression status. Our previous study demonstrated that the cisplatin-sensitive phenotype alleviated by the *BIN1* mRNA depletion (sh-BIN1) is accompanied by positive staining with Annexin-V, suggesting BIN1-induced cisplatin sensitivity is attributable, at least partly, to apoptosis [19]. We noticed that even in the presence of the wt-*TP53* gene, forced *BIN1* depletion did not elicit any morphological features of cell death by cisplatin (Figure 1C). We wondered if endogenous wt-TP53 might not be adequately phosphorylated by cisplatin because of the lack of *BIN1*. Alternatively, even if TP53 is ‘biochemically’ activated (i.e., phosphorylated) by cisplatin, we wondered if TP53 might not be ‘biologically’ activated if a wt-BIN1 expression is missing.

The phosphorylation status of serine 15 (Ser15) of human TP53 protein is believed to be the hallmark of pro-apoptotic TP53 [22,23]. Hence, we gauged the Ser15-phosphorylation of TP53 in the presence and absence of cisplatin and BIN1. We observed that, when Ser15 of TP53 was phosphorylated by BIN1 depletion, even without cisplatin, TP53 levels were visibly increased, whereas the TP53-Ser15 phosphorylation, which was caused by BIN1 loss, was markedly attenuated by KU-60019, an ATM-specific chemical inhibitor [25] (Figure 1D). This data is consistent with the fact that ATM can be activated by the lack of BIN1, a cellular inhibitor of ATM [21]. Although the levels of Ser15-phosphorylated TP53 in sh-BIN1-expressing LNCaP cells were seemingly saturated by cisplatin at 24 h post-treatment (Figure 1D), this discrepancy might be partly because of a limited quantitative range of Western blotting analysis. Consistent with this likelihood, our previous study confirmed that sh-BIN1 gradually but robustly increases endogenous TP53-dependent luciferase reporter activity in the presence of cisplatin [21]. Interestingly, even in the absence of cisplatin, the DU145 prostate cancer cell line, which was refractory to the anti-cancer activities of BIN1 and TP53 when transfected individually, became vulnerable to ectopically expressed TP53 in the DU145 cells retrovirally expressing *BIN1* (Figure S1). Since the loss of BIN1 biologically abolished cisplatin sensitivity even in the presence of TP53 (Figure 1C), we propose that BIN1 is a critical cell death-promoting component downstream of TP53. Collectively, these results suggest that TP53-dependent anti-cancer property relies, at least to some extent, on BIN1 levels.

### 2.2. BIN1 Loss Is Adequate to Initiate and Promote ATM-Dependent DNA Damage Response (DDR)-Like Signals Even in the Absence of DSBs

**Figure 2:**
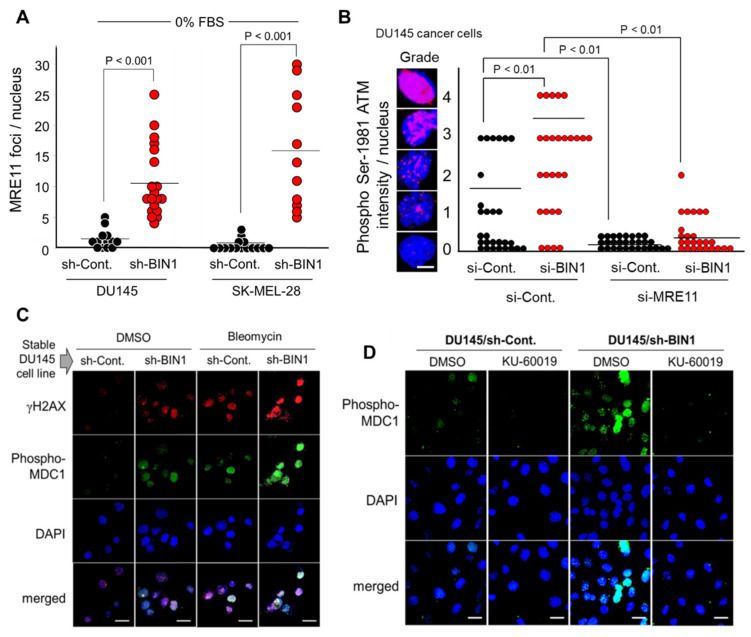
The forced BIN1 depletion increases DDR-like signals in the absence of DSBs. In situ immunofluorescence microscopy analysis demonstrated that the reduction in endogenous BIN1 by stable expression of sh-BIN1 increases (**A**) the formation of MRE11A foci even in the absence of serum; (**B**) the Ser1981-phosphorylated ATM nuclear intensity in an MRE11-dependent manner; (**C**) γH2AX foci (red) and phosphorylated MDC1 (green); and (**D**) ATM-dependent MDC1 foci (green). Bleomycin, a radiomimetic chemical, was used at 20 µg/mL for 30 min as the trigger of real DSBs [21]. KU-60019 (an ATM-selective small molecule inhibitor) was used at 3.0 µM for 72 h [21]. DMSO (dimethyl sulfoxide) was used as the vehicle control. Nuclei were counter-stained with DAPI (4′, 6-diamidino-2-phenylindole) (blue). Scale bar, 10 µm.

When DSBs occur, the MRN (MRE11A-RAD50-NBS1) complex is immediately recruited to the ends of broken dsDNA fragments, where MRN facilitates the conversion of an unphosphorylated ATM dimer (a dormant form) to a Ser1981-autophosphorylated ATM monomer (an active form) [21]. Because BIN1 suppresses ATM auto-phosphorylation, at least indirectly [21], we hypothesized that a BIN1 loss constitutively generates ATM-mediated DDR-like signals under optimal culture conditions. Using in situ immunofluorescence microscopy, we verified that forced BIN1 depletion considerably increased the MRE11A foci formation even in the absence of replicative stress due to the lack of serum (Figure 2A). Furthermore, the BIN1 depletion significantly increased ATM auto-phosphorylation in an MRE11-dependent manner (Figure 2B). The DSB-independent ATM activation by BIN1 deficiency was functionally germane because the BIN1 depletion increased ATM-dependent phosphorylation of histone variant H2AX at Ser139 (thus forming γH2AX, one of the most widely used surrogate biomarkers of DSBs). As expected, the γH2AX formation by BIN1 deficiency was efficiently enhanced by DNA-damaging agents, such as bleomycin (Figure 2C) and cisplatin [21]. Consistently, a γH2AX-binding DDR scaffold protein MDC1 (Mediator of DNA Damage Checkpoint 1, also known as Nuclear Factor with BRCT Domains 1 [NFBD1]) was concomitantly phosphorylated in the absence of BIN1 (Figure 2C) in an ATM-dependent manner (Figure 2D). These results indicate that the BIN1 loss is a natural trigger to stimulate the ATM/γH2AX-dependent conventional DDR pathways, even in the absence of DSBs.

### 2.3. MDC1 Sustains Cisplatin Resistance in BIN1-Deficient Cancer Cells Regardless of TP53

**Figure 3:**
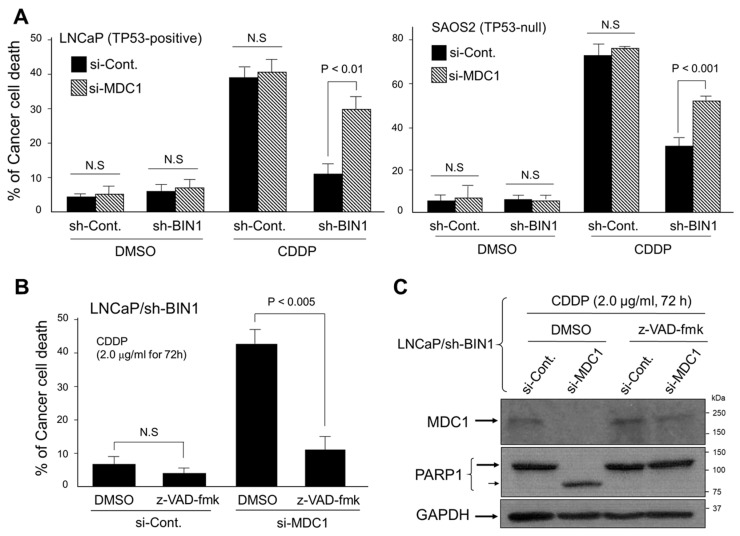
MDC1 depletion causes caspase-dependent cell death by cisplatin, thus compromising the cisplatin resistance due to a BIN1 loss. (**A**) Trypan blue exclusion assays of the cisplatin-sensitive cancer cell lines (LNCaP and SAOS2) ± sh-BIN1 in the presence and absence of cisplatin (CDDP, 2.0 µg/mL, 72 h). The scrambled sh-RNA-expression vector was used as the negative control (sh-Cont). Twenty-four hours before the treatment with cisplatin, actively growing cells were transiently transfected with si-MDC1 or si-Control RNA; (**B**) trypan blue exclusion assays demonstrated that the cisplatin sensitivity obtained by si-MDC1 was counterbalanced by z-VAD-fmk (20 µM) in the LNCaP/sh-BIN1 cells; (**C**) Western blotting analysis revealed that endogenous PARP1 protein is cleaved during cisplatin-induced apoptosis elicited by si-MDC1 in the LNCaP/sh-BIN1 cells.

Besides the role as a key DDR signal component immediately downstream of γH2AX, MDC1 is also renowned for functioning as an anti-apoptotic protein, at least in part, by physically interacting with TP53 [26]. As discussed above, BIN1 does not require TP53 for activating DNA damage-induced cell death. We, therefore, tested whether the depletion of MDC1 compromises the BIN1 loss-induced cisplatin resistance in the presence and absence of TP53. We found that, irrespective of the *TP53* gene status, the cisplatin resistance caused by BIN1 depletion was significantly attenuated by the co-transfection of *MDC1* siRNA (si-MDC1) (Figure 3A). This finding implies that BIN1-deficient cancer cells are significantly addicted to the MDC1 pathway for surviving in the presence of cisplatin.

We also observed that the cisplatin sensitivity restored by si-MDC1 in BIN1-deficient cancer cells was compromised by carbobenzoxy-valyl-alanyl-aspartyl fluoromethyl ketone (z-VAD-fmk) (20 µM), a cell-permeable pan-caspase inhibitor (Figure 3B). The blocking of si-MDC1-induced cell death by z-VAD-fmk was morphologically verified (Figure S2). As predicted, PARP1, one of the most representative cellular substrates of caspase-3 during apoptosis [27], was cleaved when si-MDC1 was transfected in BIN1-deficient cancer cells in the presence of cisplatin (Figure 3C). These results suggest that MDC1 actively counteracts caspase-dependent cell death caused by cisplatin in BIN1-deficient cancer cells in a manner independent of TP53.

### 2.4. RNF8 Protects ATM-Phosphorylated MDC1 from Caspase-3 and Sustains Cisplatin Resistance in BIN1-Deficient Cancer Cells

**Figure 4:**
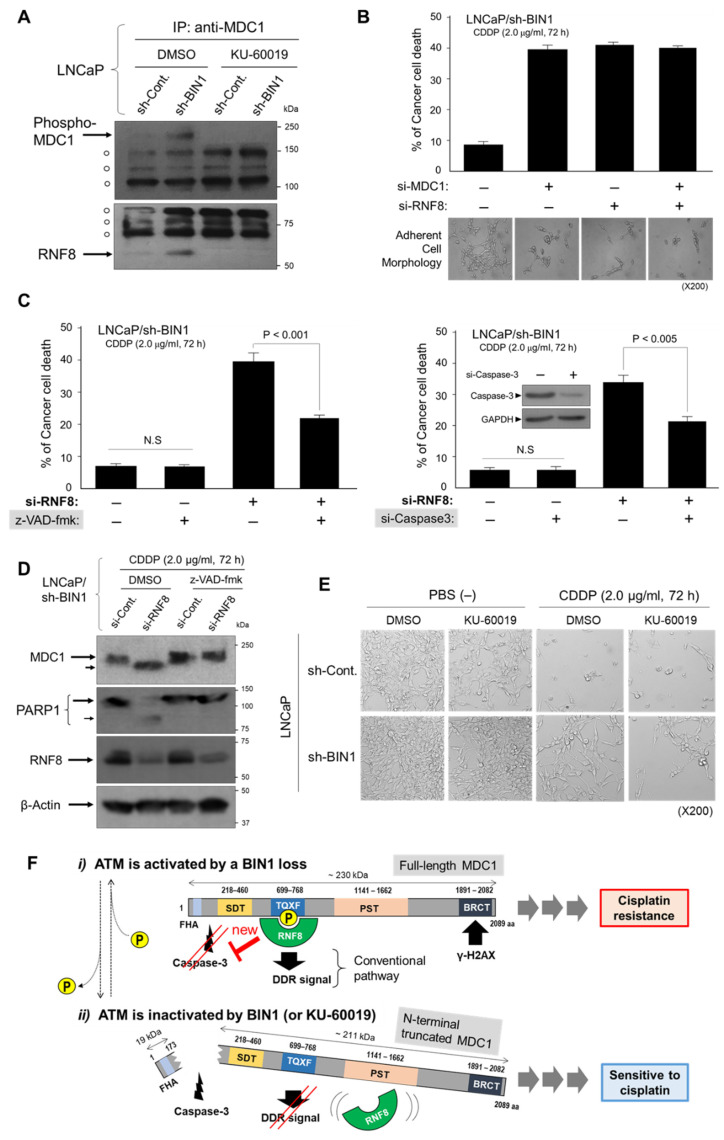
ATM-phosphorylated MDC1-RNF8 complex prevents MDC1 protein from proteolytic cleavage by caspase-3 and sustains cisplatin resistance caused by a BIN1 loss. (**A**) The cleared protein lysates immunoprecipitated with an anti-MDC1 antibody were subjected to the Western blotting analysis probed with an anti-phosphorylated-MDC1 antibody (top) and an anti-RNF8 antibody (bottom). Open circles indicate non-specific signals serving as the internal loading control; (**B**) trypan blue exclusion assays and phase-contrast microscopy after the transfection (+) of si-MDC1 or si-RNF8. The scrambled siRNAs (−) were used as the negative control; (**C**) trypan blue exclusion assays demonstrated that the si-RNF8-mediated cisplatin-induced cell death was compromised by z-VAD-fmk (20 µM) (left) and si-Caspase-3 (right); (**D**) Western blotting analysis revealed that MDC1 and PARP1 proteins are cleaved by a z-VAD-fmk-sensitive protease (most likely caspase-3 [29]) when RNF8 is depleted in the presence of cisplatin. A small arrow indicates a proteolytically cleaved product, which can be rescued by z-VAD-fmk; (**E**) KU-60019 (3.0 µM, 72 h) compromises the sh-BIN1-induced cisplatin resistance; (**F**) we propose that the MDC1-RNF8 protein complex physically protects ATM-phosphorylated MDC1 from caspase-3-dependent proteolytic cleavage in BIN1-deficient cancer cells in the presence of cisplatin. In this model, disrupting the MDC1-RNF8 complex by si-RNF8 or KU-60019 allows caspase-3 to cleave MDC1, thus inactivating the conventional MDC1-mediated DDR signals. FHA: Fork-head-associated domain, SDT: A domain required for binding to the MRN (Mre11A-Rad50-Nbs1) complex, TQXF: A domain phosphorylated by ATM, thereby mobilizing RNF8, PST: a domain that contains multiple repeats of a proline [P]-serine [S]-threonine [T] motif, BRCT: A BRCA1 C-terminal homology domain, which enables MDC1 to recognize γH2AX.

We next investigated whether MDC1, immediately downstream of γH2AX [28], determines cisplatin resistance in *BIN1*-deficient cancer cells. Once recruited by γH2AX and then phosphorylated by ATM in response to DSBs, MDC1 physically interacts with several DDR effectors, such as NBS1, 53BP1 (TP53-binding protein-1), and RNF8 (RING-finger protein 8, which is known to act as a DDR-associated E3 ubiquitin-protein ligase) [28]. There are several functional domains of MDC1, one of which, named TQXF repeat domain (T: threonine, Q: glutamine, X: any amino acid, F: phenylalanine), is the only MDC1 domain that ATM phosphorylates. More importantly, the MDC1 TQXF domain is required for the physical interaction with RNF8 [28]. As predicted, ATM-dependently phosphorylated MDC1 formed a protein complex with RNF8 only in the absence of *BIN1* under optimal culture conditions (Figure 4A). 

We next determined whether RNF8 participates in the MDC1-induced cisplatin resistance. For this purpose, RNF8 siRNA (si-RNF8) was transfected alone or co-transfected with si-MDC1 in *BIN1*-depleted cancer cells in the presence of cisplatin (2.0 µg/mL) for 72 h. The scrambled control siRNAs (si-Cont.) were used as the non-specific siRNA fillers. If ATM-phosphorylated MDC1 does not require RNF8 to mediate cisplatin resistance, we anticipated that the depletion of RNF8 alone would not change the si-MDC1-induced cisplatin sensitivity in *BIN1*-deficient cancer cells.

We found that the transfection of si-RNF8 accurately recapitulated the cisplatin sensitivity elicited by si-MDC. Moreover, the co-transfection of a half dose of each siRNA did not double the cisplatin sensitivity attained by the single transfection of either siRNA (Figure 4B). These findings indicate that RNF8 is located downstream in series rather than parallel to the MDC1-mediated cisplatin resistance in BIN1-deficient cancer cells. As expected, the cisplatin sensitivity induced by si-RNF8 was significantly compromised by a pan-caspase inhibitor z-VAD-fmk (20 µM) or si-Caspase-3 in BIN1-deficient cancer cells (Figure 4C). Although MDC1 was known to be proteolytically cleaved by caspase-3 under various pro-apoptotic conditions [29], it was unclear whether RNF8 positively or negatively regulates the MDC1 cleavage by caspase-3. In this experiment, PARP1 was used as the positive control for caspase-3-dependent proteolytic cleavage. When si-RNF8 was transfected in the presence of cisplatin, MDC1 (~230 kDa) was cleaved by a z-VAD-fmk-sensitive protease (most likely caspase-3), thus producing a slightly truncated MDC1 polypeptide (Figure 4D). Moreover, the ATM inhibitor KU-60019, which dissociated the MDC1-RNF8 complex (see above, in Figure 4A), rendered BIN1-deficient (thus, cisplatin-resistant) cancer cells vulnerable to cisplatin (Figure 4E). Collectively, these results suggest that RNF8 physically binds to and protects ATM-phosphorylated MDC1 from caspase-dependent proteolytic cleavage in BIN1-deficient cancer cells. Since the formation of the ATM-dependent MDC1-RNF8 complex is associated with cancer-cell survival in the presence of cisplatin, we propose that ATM inactivation by an ATM inhibitor, which destabilizes the MDC1-RNF8 interaction, would be a possible therapeutic option to restore cisplatin sensitivity even in BIN1-deficient (i.e., cisplatin-resistant) cancer cells (Figure 4F).

### 2.5. Long-Term and Repeated Exposure to Cisplatin Spontaneously Recapitulates a BIN1 Loss and Consequent RNF8-Dependent Cisplatin Resistance

Forced BIN1 depletion by sh-BIN1 helped us reveal that BIN1 and RNF8 are oppositely involved in cisplatin sensitivity (see above, in Figure 4). Indeed, these findings provided an exciting clue to better understanding the underlying mechanism of cisplatin resistance, but we could not rule out that this new RNF8-controlling mechanism might be an artificial cellular response due to a sudden and enforced reduction in endogenous BIN1. To mimic the effects of long-term and repeated exposure to cisplatin ultimately leading to cisplatin resistance, we established a cisplatin-resistant cancer-cell model system in vitro. To do this, we discontinuously and repeatedly exposed proliferating cisplatin-sensitive cancer cells to gradually increasing doses of cisplatin in vitro every other week for more than two months. As shown in Figure 1B (see above), transfected sh-BIN1 prominently compromised the sensitivities of LNCaP (prostate cancer) and U2OS (osteosarcoma) cell lines to cisplatin. So, we used these two independent cisplatin-sensitive cancer cell lines as the parental cell lines for developing the natural cisplatin-resistant model cell systems in vitro. 

**Figure 5:**
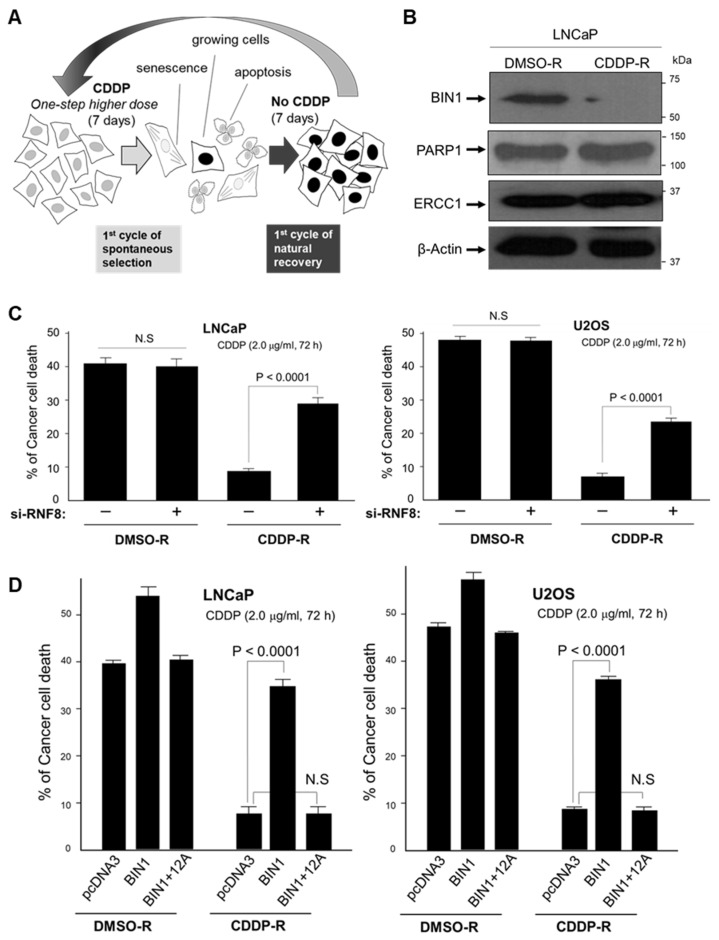
Spontaneous BIN1 loss and following RNF8-dependent cisplatin resistance emerge after long-term cisplatin exposure. (**A**) The establishment of spontaneous cisplatin (CDDP)-Resistant cancer cell lines (CDDP-R). The same parental cell line was incubated with 1/100 [*v*/*v*] of DMSO Repeatedly (DMSO-R) as the vehicle control cell line. The concentration of cisplatin of each treatment cycle was gradually increased from 0.5, 1.0, 2.0 to 4.0 µg/mL; (**B**) Western blotting analysis of *BIN1*, PARP1, and ERCC1 in the CDDP-R and DMSO-R cell lines. An open circle indicates a non-specific signal; (**C**) transiently transfected si-RNF8 restored cisplatin sensitivity in the CDDP-R cancer cell lines but not in the DMSO-R cell lines; (**D**) naturally acquired cisplatin resistance after long-term cisplatin exposure was balanced by transiently transfected *BIN1* cDNA but not *BIN1* *+ 12A* cDNA.

To judge whether a cancer cell line is sensitive to cisplatin, we regularly use 2.0 µg/mL of cisplatin for 72 h [19] (see Figure 1B). In order to render parental LNCaP and U2OS cells stably resistant to cisplatin, these cell lines were incubated in an optimal growth medium containing increasing doses of cisplatin at 0.5, 1.0, 2.0, and 4.0 µg/mL every other week. As the negative vehicle control, we used the same amount (1/100 [*v*/*v*]) of dimethyl sulfoxide (DMSO) repeatedly (DMSO-R). Before we proceeded to the next higher dose of cisplatin, the cancer cells, which had somehow managed to survive or grow in the presence of a one-step lower concentration of cisplatin during the former week, were cultured without cisplatin for another week. During this interval without cisplatin, those cancer cells damaged by cisplatin were expected to be recovered. If the cancer cells spontaneously acquired cisplatin resistance (or, if a minor cancer stem-cell-like population is gradually expanded), those cells should actively grow even in the presence of 2.0 µg/mL cisplatin at least one week. In order to avoid losing the newly acquired cisplatin-resistant phenotype, the stable cisplatin-resistant (CDDP-R) cancer cell lines were maintained in the presence of 1.0 µg/mL cisplatin (Figure 5A).

After the long-term exposure to cisplatin, endogenous BIN1 levels markedly reduced (Figure 5B). We initially expected that long-term cisplatin exposure might reveal a new mechanism for cisplatin resistance other than BIN1 depletion, so we were surprised to see this naturally recapitulated BIN1 loss, which coincided with the result by sh-BIN1. PARP1 and Excision Repair Cross-Complementation Group 1 (ERCC1) proteins are well known to be crucial to mediate the NER pathway vital for repairing platinum-DNA adducts [19,30,31], but we saw no significant increase in either NER-related protein. This result implies that BIN1 could be critically involved in inhibiting the NER pathway [19]. Alternatively, an NER-independent DSB repair mechanism, such as the ATM-dependent MDC1-RNF8 pathway (as discussed above), might be activated by spontaneous BIN1 loss before the levels of PARP1 or ERCC1 are visibly increased by cisplatin. Notably, transfection of si-RNF8 significantly increased cisplatin sensitivity in the LNCaP^CDDP-R^ and U2OS^CDDP-R^ cell lines (Figure 5C).

Furthermore, ectopically expressed *BIN1* cDNA re-established cisplatin sensitivity in the spontaneously established CDDP-R cancer cell lines, whereas *BIN1* *+ 12A* cDNA transfection did not (Figure 5D). These results suggest that the natural reduction of endogenous BIN1 levels after long-term cisplatin exposure is not one of the biochemical consequences accompanying cisplatin resistance but a likely initiating factor of the spontaneous acquisition of cisplatin resistance. Therefore, we determined when and how the *BIN1* gene expression is suppressed in the following experiments while cancer cells acquire cisplatin resistance.

### 2.6. Inhibition of PARP Activity Reverses Hyperactivated MYC-Dependent BIN1 Gene Repression, Thereby Restoring Cisplatin Sensitivity in the CDDP-R Cell Lines

**Figure 6:**
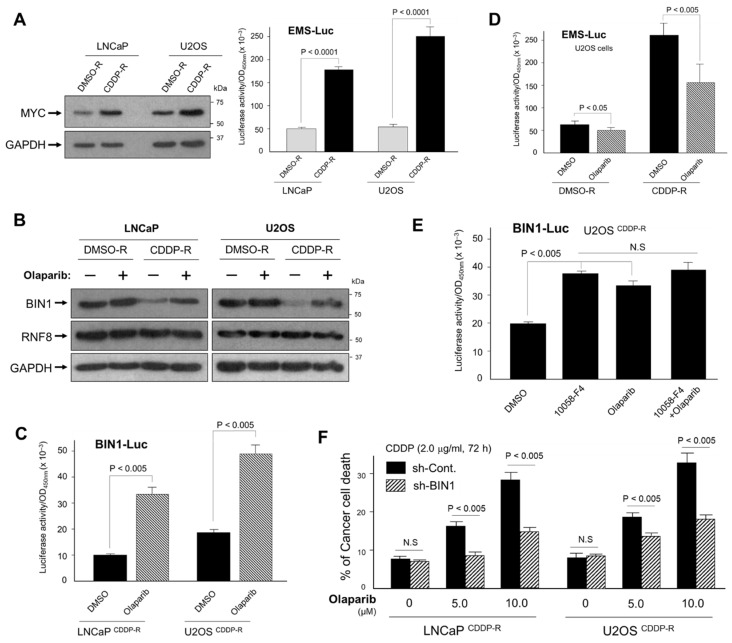
Coordinated actions of MYC and PARP1 play a critical role in BIN1 reduction and following cisplatin resistance in the CDDP-R cell lines. The increased activity of the neoplastic MYC transcription factor depends greatly on increased PARP activity in the CDDP-R cancer cells, thus repressing *BIN1* promoter activity and conversely increasing cisplatin resistance. (**A**) Western blotting analysis (left) and an EMS-luciferase reporter assay driven by the MYC-sensitive promoter sequence EMS (E-box multiple sites) (right) in the LNCaP^CDDP-R^ and U2OS^CDDP-R^ cell lines. The LNCaP^DMSO-R^ and U2OS^DMSO-R^ cell lines were used as the negative control; (**B**) Western blotting analysis of BIN1 and RNF8 ± olaparib in the indicated cell lines; (**C**) the human *BIN1* gene promoter activity was monitored by the BIN1-Luc reporter ± olaparib in the indicated cell lines; (**D**) the EMS-Luc reporter assay in the U2OS^CDDP-R^ and U2OS^DMSO-R^ cell lines ± olaparib (5.0 µM, 48 h); (**E**) the effects of 10058-F4 (a small molecule MYC inhibitor: 16 µM, 48 h) [19] and olaparib (5.0 µM, 48 h) [20] on the BIN1-Luc reporter activity in the U2OS^CDDP-R^ cell line; (**F**) transiently transfected sh-BIN1 significantly reversed olaparib/cisplatin-induced cancer-cell death in the LNCaP^CDDP-R^ and U2OS^CDDP-R^ cell lines.

It has been documented that MYC promotes the resistance of various cancer cells to cisplatin [19,32,33,34,35,36,37,38], but precisely how this oncoprotein increases cisplatin resistance is unclear. Because BIN1 was initially identified as a cellular MYC inhibitor [9,10,13], we were curious about the status of endogenous MYC expression and its activity in the CDDP-R cell systems, where endogenous BIN1 protein was remarkably reduced (see Figure 5B). To monitor endogenous MYC activity, we transiently transfected the E-box multiple-sites (EMS)-driven luciferase reporter vector (EMS-Luc), which can be activated exclusively by the MYC family of transcription factors [13]. We found that endogenous MYC levels and the transcriptional activity were more robustly increased in LNCaP^CDDP-R^ and U2OS^CDDP-R^ cell lines than the matched DMSO-R cell lines (Figure 6A). 

Besides MYC inhibition [9,10,13], BIN1 physically binds to the auto-modification domain of PARP1 and suppresses its catalytic activity [19]. Thus, we next determined whether olaparib, a small molecule PARP1/2 inhibitor, restores BIN1 levels in the CDDP-R cancer cell lines. The amount of RNF8 was not altered by either cisplatin or olaparib, whereas the disappearance of BIN1 protein after long-term exposure to cisplatin was counterbalanced by olaparib only in the CDDP-R cell lines (Figure 6B). As expected, olaparib robustly activated the *BIN1* gene promoter in these CDDP-R cancer cells (Figure 6C). Interestingly, we noticed that cellular poly(ADP-ribosylation) (or PARylation) was visibly increased in the CDDP-R cells (Figure S3), presumably due to progressive production of SSBs and DSBs by cisplatin. This result indicates that cancer cells gradually became dependent on their PARP activity in order to survive in the presence of cisplatin. Of note, endogenous MYC activity monitored by the co-transfected EMS-Luc reporter gene was prominently up-regulated in the CDDP-R cells and was inhibited by olaparib, whereas the EMS-Luc was not so activated in the matched DMSO-R cells (Figure 6D). Thus, we hypothesized that, in spontaneously cisplatin-resistant cancer (CDDP-R) cell lines, endogenous PARP1 activity is required for stimulating MYC activities.

Interestingly, the small molecule MYC-specific inhibitor 10058-F4 [39,40] was sufficient to increase the BIN1-Luc activity in the CDDP-R cells. In contrast, the combined use of olaparib and 10054-F4 did not double the BIN1-Luc activity and merely displayed almost the same upsurge as either inhibitor alone (Figure 6E). We, therefore, concluded that PARP1 and MYC do not independently attenuate the BIN1-Luc activity but are located on the same signal pathway leading to the *BIN1* gene repression in the CDDP-R cells. As predicted, re-establishing the cisplatin sensitivity in the CDDP-R cells was dose-dependently achieved by olaparib and was significantly curbed by co-transfected sh-BIN1 (Figure 6F). We propose that, once cancer cells spontaneously acquire cisplatin resistance, we can use a PARP inhibitor (instead of a still-controversial MYC inhibitor) to inhibit oncogenic MYC activity so that we can recover endogenous *BIN1* expression and accompanying cisplatin sensitivity.

## 3. Discussion

Since the original discovery of cisplatin as a unique chemical compound that suppresses bacterial cell division [1,2] and human cancer-cell growth in vitro and in vivo nearly five decades ago [3,4], its illustrious therapeutic efficacy has been documented in various human cancers [5,6,7]. However, the emergence of cisplatin-resistant cancer cells remains an unresolved and continued clinical issue today. To improve the poor prognosis of the patients with cisplatin-refractory cancer, identifying an Achilles’ Heel (i.e., a vulnerable fatal weakness) of cisplatin-resistant cancer cells has continued to be a central interest in basic and clinical cancer research. In general, the acquisition of cisplatin resistance is associated with advanced or late stages of human cancers. Although the pro-apoptotic BIN1 tumor suppressor facilitates cisplatin sensitivity but is missing in various human cancer cells in vitro [18,19,20,21], little is known about whether (and how) BIN1 could be restored in cisplatin-refractory cancer cells. Moreover, identifying downstream effectors activated by BIN1 deficits would be crucial for better understanding cisplatin resistance. 

**Figure 7:**
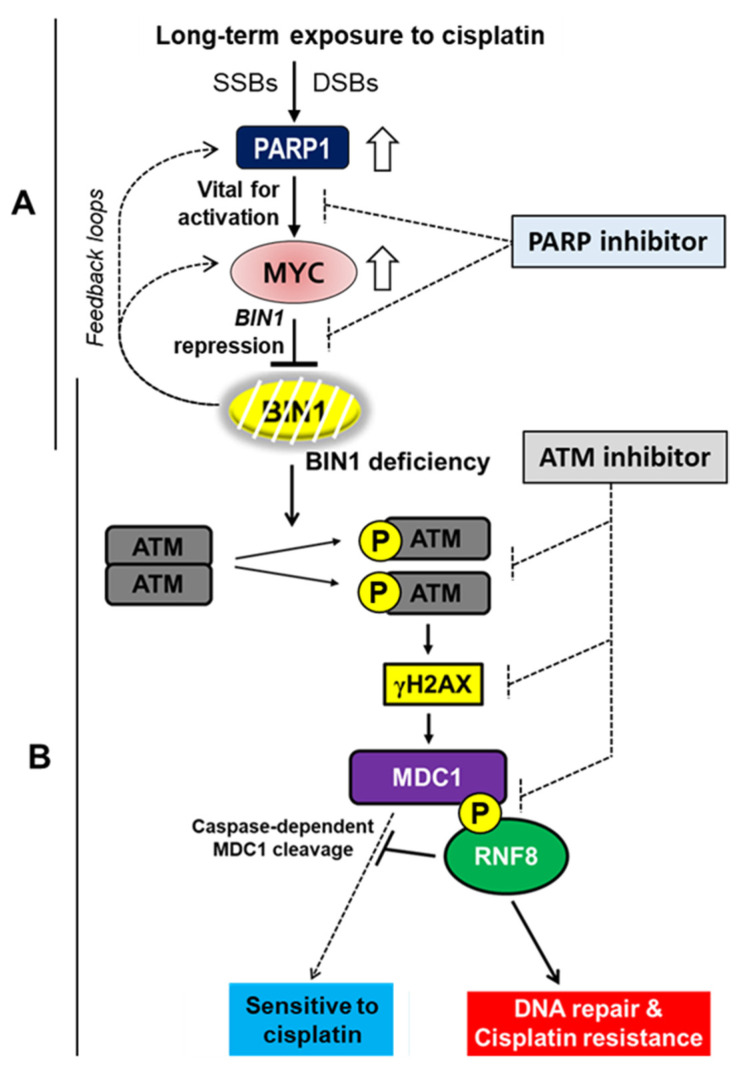
New synthetic lethal signaling cascades by co-using PARP and ATM inhibitors to eradicate the resistance to cisplatin. (**A**) During DNA replication, platinum-DNA adducts increase SSBs and DSBs, which automatically activate PARP1, one of the most immediate-early nuclear enzymes responsible for maintaining genome stability [19,45]. Subsequently, activated PARP1 assists MYC to repress *BIN1* promoter activity (this study). Because reduced BIN1 automatically activates endogenous PARP1 [19] and MYC activities [9,10,13], BIN1 deficiency forms unique feedback loops to infinitely repress the *BIN1* promoter activity by activating PARP1 and MYC. Instead of directly targeting neoplastic MYC transcription factor, a PARP inhibitor, which is currently approved for clinical study for human cancers in combination with ‘BRCAness,’ could be an alternative strategy to attenuate deregulated MYC activity in spontaneously cisplatin-resistant cancer cells; (**B**) by physically releasing the E2F1 nuclear protein [21] (omitted here), a BIN1 loss facilitates ATM auto-phosphorylation, thereby promoting γH2AX formation and following ATM-dependent MDC1 phosphorylation [21,28]. Accordingly, RNF8 physically binds to ATM-phosphorylated MDC1, thus prohibiting caspase-3-induced proteolytic cleavage of MDC1 [29]. We propose that the ATM-dependent formation of the MDC1-RNF8 complex serves as a watershed to interfere with cisplatin sensitivity, thereby facilitating DSB repair and subsequent cisplatin resistance.

Mitchels et al. (2013) reported that most cisplatin-resistant cancer cells develop an addiction to endogenous PARP1 activity [41] and that PARP inhibition provokes apoptosis by cisplatin regardless of the status of TP53 [42]. Nonetheless, how PARP1 is hyperactivated frequently in cisplatin-resistant cancer cells and how PARP inhibition elicits TP53-independent cancer-cell death remained obscure. One of the critical findings of our current study is that a small molecule PARP inhibitor (i.e., olaparib) suppresses the activity of neoplastic MYC transcription factor, especially in cancer cells spontaneously acquired cisplatin resistance. In general, MYC is overexpressed or deregulated in many human malignancies, so neoplastic MYC activity has been suggested to be an ideal target for cancer chemotherapy [43]. However, the development and clinical use of the current small molecule MYC inhibitor are still controversial mainly because of the lack of high MYC specificity and potential off-target effects thereof. Of course, a PARP inhibitor is not originally designed to inhibit MYC activity per se, but our finding suggests that inhibiting PARP activity curbs neoplastic MYC activity only when cancer cells acquire cisplatin resistance (see Figure 6D). BIN1 protein itself is a cellular inhibitory factor for MYC and PARP1 [9,10,13,19] and activates cisplatin sensitivity irrespective of TP53 (see Figure 1). Therefore, the prolonged exposure to cisplatin, which gradually accumulates SSBs and DSBs, thus stimulating PARP1 activity (Figure S3), likely promotes MYC-dependent *BIN1* repression, thereby forming a unique positive feedback loop to decrease *BIN1* expression further (Figure 7A). Considering the ongoing clinical use of a PARP inhibitor, we propose that shutting down the ‘PARP1-MYC-*BIN1* loss’ feedback loop by a PARP inhibitor would be a feasible strategy to restore endogenous *BIN1* expression and accompanying TP53-independent cisplatin sensitivity. It will be interesting to determine how neoplastic MYC, which does not remarkably require PARP activity in cisplatin-sensitive (i.e., early-stage) cancer cells, considerably depends on the PARP activity in cisplatin-resistant cancer cells. 

Cancer patients with impaired DDR pathways (including the ATM pathways) generally respond better to cisplatin [44]. With this regard, another crucial mechanism we identified in this study leading to BIN1 loss-mediated cisplatin resistance is the ATM-dependent ‘MDC1-RNF8’ signaling pathways. BIN1 attenuates ATM auto-phosphorylation, at least indirectly [21]. Thus, forming an ATM-phosphorylated MDC1-RNF8 protein complex is a critical determinant of cisplatin resistance in cancer cells defective in *BIN1* expression. In response to activated ATM due to BIN1 deficiency, MDC1 is constitutively phosphorylated by ATM and transduces the ATM-dependent DDR signals even before DSBs happen. Solier and Pommier (2011) reported that MDC1 is cleaved by caspase-3 in response to various pro-apoptotic stimuli [29]. This finding was the first evidence that the MDC1-RNF8 DDR pathway for cell survival is inversely interconnected to DNA damage-induced apoptosis. However, it remained unclear how the MDC1 cleavage by caspase-3 could be prohibited in cisplatin-resistant cancer cells. Our study demonstrated that RNF8 E3 ubiquitin-protein ligase, which is known to bind ATM-phosphorylated MDC1 to promote histone ubiquitylation nearby DSB lesions for promoting DSB repair signals [28], actually protects ATM-phosphorylated MDC1 from caspase-3-dependent cleavage in BIN1-deficient cancer cells, thereby sustaining cisplatin resistance. Therefore, if ATM activity is constitutively attenuated by endogenous BIN1 (a natural ATM silencer) or an ATM-specific small molecule inhibitor [21], the MDC1-RNF8 protein complex could be dissociated, which then allows caspase-3 to cleave MDC1 (see Figure 4) for eliciting cisplatin sensitivity. Therefore, besides a PARP inhibitor, an ATM-specific inhibitor would be another attractive therapeutic approach for eliminating cisplatin-refractory cancer cells if combined with cisplatin (Figure 7B).

‘BRCAness’ represents a genetic (or even a phenotypic) impairment of the hereditary breast/ovarian cancer susceptibility *BRCA1/2* genes [45]. The synthetic lethality elicited by a PARP inhibitor monotherapy in cancers with ‘BRCAness’ has brought a striking impact on experimental and clinical cancer therapeutics [46]. However, as time passed, second mutations in the *BRCA2* gene that reshifted the open reading frames of an already frame-shifted *BRCA2* gene were identified [47]. This double-mutation model indicates that some *BRCA1/2*-mutated cancers could express wt-BRCA1/2-like full-length proteins and, thus, become refractory to a PARP inhibitor monotherapy [47]. Compared with the high frequency of ‘BRCAness’ in hereditary breast/ovarian cancer patients, sporadic cancers do not usually display typical ‘BRCAness’ except for triple-negative breast cancer exhibiting a ‘BRCAness’-like phenotype [48]. Hence, the potential population of cancer patients who benefited from conventional PARP inhibitor/‘BRCAness’-dependent synthetic lethality chemotherapy is extremely restricted. This situation explains why a ‘BRCAness’-independent synthetic lethal mechanism to increase the cancer susceptibility to a PARP inhibitor (or other chemotherapy) has been long-awaited. Indeed, probably because ‘BRCAness’ and cisplatin cause genome instability of cancer cells, PARP inhibitors were occasionally applied to treat cisplatin-sensitive cancers irrespective of ‘BRCAness’ [44,45]. However, a synthetic lethal target collaborating with PARP inhibition and cisplatin has not been fully elucidated. Under this circumstance, it has been unimaginable to use a PARP inhibitor in even more advanced cisplatin-refractory cancers. 

Similar to the *BIN1* gene, the *MDC1*, *RNF8*, *PARP1*, *MYC*, and *ATM* genes are not as frequently mutated as the *TP53* gene in most human cancers (Figure S4). If combined with an ATM inhibitor (which shuts down the ‘MDC1-RNF8’-mediated DDR pathway—see Figure 7B), a PARP1 inhibitor (which rescues endogenous *BIN1* expression by cutting down the ‘PARP1–MYC’ feedback loop—see Figure 7A) will generate a new ‘BRCAness-independent’ synthetic lethal effect. Therefore, a potent anti-cancer chemotherapeutic protocol to co-use cisplatin (or other platinum compounds, such as carboplatin and oxaliplatin) with PARP and ATM inhibitors clinically deserves establishing as it may systemically restore the susceptibility to cisplatin in platinum-refractory recurrent cancers.

## 4. Materials and Methods

### 4.1. Mammalian Cell Lines 

All mammalian cell lines used in this study were obtained from the American Type Culture Collection (ATCC, Manassas, VA, USA) or have been described previously [18,19,20,21], and they were maintained in an appropriate culture medium supplemented with 2.0 mM L-glutamine and 10% fetal bovine serum in 5% CO2 at 37 °C according to the vendor’s instructions.

### 4.2. Chemicals, Antibodies, and sh-RNAs/si-RNAs

The chemicals, antibodies, and sh-RNAs/si-RNAs used in this study are listed in the Supplementary Materials.

### 4.3. Plasmid DNAs

The pcDNA3 expression vectors for human *BIN1* (pcDNA3–BIN1) and *BIN1*+12A cDNAs (pcDNA3–BIN1+12A), the EMS (E-box multiple sites) promoter-driven luciferase reporter vector (EMS-Luc), and the human *BIN1* gene promoter-driven luciferase reporter vector (BIN1-Luc) used in this study have been described previously [13,14,15].

### 4.4. DNA and siRNA Transfection

The FuGENE-6 (Promega, Madison, WI, USA) and X-tremeGENE (Millipore-Sigma, St. Louis, MO, USA) reagents were used for transfecting plasmid DNA and siRNA, respectively, according to the vendor’s instructions.

### 4.5. Recombinant Lentivirus Infection

The human *BIN1* sh-RNA (sh-*BIN1*)–expressing lentivirus plasmid DNA (sc-29804-SH, Santa Cruz Biotechnology, Dallas, TX, USA) was co-transfected in the packaging cell line HEK293T with the lentivirus packaging vector psPAX2 (Addgene, Cambridge, MA, USA) and the lentivirus envelope–expressing vector pMD2.G (Addgene). The scrambled sh-RNAs (sc-108,060, Santa Cruz Biotechnology) were used as the negative control (sh-control). The packaging culture supernatant was harvested at 72 h post-transfection and freshly overlaid on the host cell line for 72 h in the presence of puromycin. All drug-resistant colonies were collected and subjected to biochemical and cell-based analysis. 

### 4.6. Immunoprecipitation/Western Blot Analysis

With gentle rocking, approximately 2.0 mg of precleared mammalian protein lysates were incubated with 1.5 µg of an immunoprecipitation (IP) antibody at 4 °C for 4 h. Purified pre-immune mouse IgG (Pierce, Rockford, IL, USA) was a negative control. The immunoprecipitated protein complexes or precleared lysates were resolved by sodium-dodecyl-sulfate polyacrylamide gel electrophoresis (SDS–PAGE) and transferred to a nitrocellulose membrane for 1 h at 100 V in Towbin buffer (192 mM glycine, 20% methanol, and 25 mM Tris–HCl, pH 8.3). Membranes were blocked using 5% non-fat skim milk in PBST (PBS containing 0.1% Tween 20) overnight at 4 °C with gentle rocking, then hybridization with the appropriate primary and secondary antibodies. Glyceraldehyde-3-phosphate dehydrogenase (GAPDH) or β-actin was used as the internal protein loading control. 

### 4.7. Luciferase Reporter Assay

Approximately 1 × 10^6^ cells per 10-cm tissue culture dish were harvested after the treatment with indicated experimental conditions and subjected to luciferase assays. Luciferase assays were performed as described previously [18,19,20,21]. As an internal transfection control to normalize the transfection efficiency, a β-galactosidase expression vector (pcDNA3-β-Gal) was co-transfected with a one-tenth quantity of a luciferase reporter vector. 

### 4.8. Trypan Blue Exclusion Assay

Cell viability was determined by trypan blue exclusion assay as described previously [18,19,20,21]. Briefly, approximately 1 × 10^6^ cells per a 10-cm tissue culture dish after the treatment with indicated experimental conditions were trypsinized. Trypsinized cells were gently suspended in PBS at r.t., then 10 µL of the cell suspension and 10 µL of trypan blue solution (GE Healthcare—HyClone Laboratories Inc., Logan, UT, USA) were mixed, and 10 µL of this mixture were immediately applied for cell counting using a hemocytometer to record the number of blue-stained (dead) cells and non-stained (alive) cells.

### 4.9. In Situ Immunofluorescence Microscopy

The cells fixed in 3.7% formaldehyde, permeabilized with 0.25% Triton X-100 in PBS, were soaked in 3% bovine serum albumin (BSA) for blocking at r.t. for 1 h. The primary antibody was diluted in PBS and was applied to the cells at r.t. for 1 h. The secondary antibodies coupled to Alexa Fluor 488 (Molecular Probes, Eugene, OR, USA) in blocking buffer were incubated at r.t. in the dark. We routinely used 4′, 6-diamidino-2-phenylindole (DAPI) for cell nuclear counterstaining (Sigma-Aldrich, St. Louis, MO, USA). The cell images were monitored with a Leica DM 5500 fluorescence microscope (Leica Microsystems, Buffalo Grove, IL, USA) and recorded with a digital camera, CoolSNAP HQ2 (Photometrics, Tucson, AZ, USA).

### 4.10. Statistical Analysis

All cell-based assays described above were repeated separately, at least three times. The raw data are presented as means ± standard errors. Statistical significance was determined with Student’s t-test, and *p*-values less than 0.05 were considered statistically significant.

## 5. Conclusions

Neoplastic MYC transcription factor promotes malignant phenotypes of human cancers, including cisplatin resistance, but the signaling mechanisms through which MYC increases cisplatin resistance are not fully understood. Moreover, a continued and potential challenging aspect of the anti-MYC research is the lack of a highly specific small-molecule MYC inhibitor that selectively inactivates one of the targeting MYC functions, such as cisplatin resistance. MYC is essential for sustaining various normal cellular functions, such as metabolism, so complete inactivation of MYC would not be ideal. Therefore, one of the current tendencies of the anti-MYC research is to identify MYC downstream effectors, which play a critical role in combination with other chemotherapy, such as PARP inhibitors [49]. Alternatively, irrespective of MYC, a crucial signaling component of homologous recombination other than BRCA1/2, such as RAD51 and CtIP, could also be a potential therapeutic target combined with a PARP inhibitor [50]. Our study has identified two novel MYC effector pathways that promote cisplatin resistance cooperatively. One is the PARP1-MYC pathway for BIN1 reduction, which spontaneously emerges when cancer cells become cisplatin-resistant, and the other is the ATM-phosphorylated MDC1-RNF8 signaling pathway by a BIN1 loss. Therefore, instead of directly targeting neoplastic MYC, our study theoretically justifies clinically approved PARP and ATM inhibitors that can be co-used to re-establish platinum sensitivity even in platinum-refractory cancer cells.

## Data Availability

Data available in a publicly accessible repository.

## Supplementary Materials

The following are available online at https://www.mdpi.com/article/10.3390/ijms222413324/s1.

## Abbreviations

ATM: ataxia-telangiectasia mutated serine/threonine kinase; BIN1: bridging integrator-1; CDDP: cis-diammine-dichloro-platinum (or cisplatin); DAPI: 4’, 6-diamidino-2-phenylindole; DDR: DNA damage response; DSB: double-stranded DNA break; GAPDH: glyceraldehyde-3-phosphate dehydrogenase; γH2AX: phosphorylated serine 139 in histone H2AX (human and mouse); MDC1: mediator of DNA damage checkpoint protein 1; PARP1: poly(adenosine diphosphate [ADP]-ribose) polymerase 1; RNF8: Ring Finger Protein-8; sh-RNA: short hairpin RNA; si-RNA: small interfering RNA; SSB: single-stranded DNA break

## References

[1] Rosenberg B., Van Camp L., Krigas T. (1965). Inhibition of cell division in Escherichia Coli by electrophoresis products from a platinum electrode. Nature.

[2] Rosenberg B., Van Camp L., Trosko J.E., Mansour V.H. (1965). Platinum compounds: A new class of potent antitumour agents. Nature.

[3] Einhorn L.H., Donohue J. (1977). Cis-diamminedichloroplatinum, vinblastine, and bleomycin combination chemotherapy in disseminated testicular cancer. Ann. Intern. Med..

[4] Einhorn L.H., Williams S.D. (1979). The role of cis-platinum in solid-tumor therapy. N. Engl. J. Med..

[5] Rottenberg S., Disler C., Perego P. (2021). The rediscovery of platinum-based cancer therapy. Nat. Rev. Cancer.

[6] Ghosh S. (2019). Cisplatin: The first metal based anti-cancer drug. Bioorg. Chem..

[7] Duan M., Ulibarri J., Liu K.J., Mao P. (2021). Role of nucleotide excision repair in cisplatin resistance. Int. J. Mol. Sci..

[8] Cleaver J.E. (2005). Cancer in xeroderma pigmentosum and related disorders of DNA repair. Nat. Rev. Cancer.

[9] Sakamuro D., Elliott K.J., Wechsler-Reya R., Prendergast G.C. (1996). BIN1 is a novel MYC-interacting protein with features of a tumour suppressor. Nat. Genet..

[10] Elliott K., Sakamuro D., Basu A., Du W., Wunner W., Staller P., Gaubatz S., Zhang H., Prochownik E., Eilers M. (1999). Bin1 functionally interacts with Myc and inhibits cell proliferation via multiple mechanisms. Oncogene.

[11] DuHadaway J.B., Sakamuro D., Ewert D.L., Prendergast G.C. (2001). Bin1 mediates apoptosis by c-Myc in transformed primary cells. Cancer Res..

[12] Pineda-Lucena A., Ho C.S., Mao D.Y., Sheng Y., Laister R.C., Muhandiram R., Lu Y., Seet B.T., Katz S., Szyperski T. (2005). A structure-based model of the c-Myc/Bin1 protein interaction shows alternative splicing of Bin1 and c-Myc phosphorylation are key binding determinants. J. Mol. Biol..

[13] Kinney E.L., Tanida S., Rodrigue A.A., Johnson J.K., Tompkins V.S., Sakamuro D. (2008). Adenovirus E1A oncoprotein liberates c-Myc activity to promote cell proliferation through abating Bin1 expression via an Rb/E2F1-dependent mechanism. J. Cell. Physiol..

[14] Wechsler-Reya R., Sakamuro D., Zhang J., Duhadaway J., Prendergast G.C. (1997). Structural analysis of the human BIN1 gene. Evidence for tissue-specific transcriptional regulation and alternate RNA splicing. J. Biol. Chem..

[15] Lundgaard G.L., Daniels N.E., Pyndiah S., Cassimere E.K., Ahmed K.M., Rodrigue A., Kihara D., Post C.B., Sakamuro D. (2011). Identification of a novel effector domain of BIN1 for cancer suppression. J. Cell. Biochem..

[16] Ge K., Duhadaway J., Sakamuro D., Wechsler-Reya R., Reynolds C., Prendergast G.C. (2000). Losses of the tumor suppressor BIN1 in breast carcinoma are frequent and reflect deficits in programmed cell death capacity. Int. J. Cancer.

[17] Ge K., Minhas F., Duhadaway J., Mao N.C., Wilson D., Buccafusca R., Sakamuro D., Nelson P., Malkowicz S.B., Tomaszewski J. (2000). Loss of heterozygosity and tumor suppressor activity of Bin1 in prostate carcinoma. Int. J. Cancer.

[18] Cassimere E.K., Pyndiah S., Sakamuro D. (2009). The c-MYC-interacting pro-apoptotic tumor suppressor BIN1 is a transcriptional target for E2F1 in response to DNA damage. Cell. Death Differ..

[19] Pyndiah S., Tanida S., Ahmed K.M., Cassimere E.K., Choe C., Sakamuro D. (2011). c-MYC suppresses BIN1 to release poly (ADP-ribose) polymerase 1: A mechanism by which cancer cells acquire cisplatin resistance. Sci. Signal.

[20] Kumari A., Iwasaki T., Pyndiah S., Cassimere E.K., Palani C.D., Sakamuro D. (2015). Regulation of E2F1-induced apoptosis by poly (ADP-ribosyl) ation. Cell. Death Differ..

[21] Folk W.P., Kumari A., Iwasaki T., Pyndiah S., Johnson J.C., Cassimere E.K., Abdulovic-Cui A.L., Sakamuro D. (2019). Loss of the tumor suppressor BIN1 enables ATM Ser/Thr kinase activation by the nuclear protein E2F1 and renders cancer cells resistant to cisplatin. J. Biol. Chem..

[22] Vogelstein B., Kinzler K.W. (2004). Cancer genes and the pathways they control. Nat. Med.

[23] Pfister N.T., Prives C. (2017). Transcriptional regulation by wild-type and cancer-related mutant forms of p53. Cold Spring Harb. Perspect. Med..

[24] Ge K., DuHadaway J., Du W., Herlyn M., Rodeck U., Prendergast G.C. (1999). Mechanism for elimination of a tumor suppressor: Aberrant splicing of a brain-specific exon causes loss of function of Bin1 in melanoma. Proc. Natl. Acad. Sci. USA.

[25] Golding S.E., Rosenberg E., Valerie N., Hussaini I., Frigerio M., Cockcroft X.F., Chong W.Y., Hummersone M., Rigoreau L., Menear K.A. (2009). Improved ATM kinase inhibitor KU-60019 radiosensitizes glioma cells, compromises insulin, AKT and ERK prosurvival signaling, and inhibits migration and invasion. Mol. Cancer Ther..

[26] Nakanishi M., Ozaki T., Yamamoto H., Hanamoto T., Kikuchi H., Furuya K., Asaka M., Delia D., Nakagawara A. (2007). NFBD1/MDC1 associates with p53 and regulates its function at the crossroad between cell survival and death in response to DNA damage. J. Biol. Chem..

[27] Soldani C., Scovassi A.I. (2002). Poly (ADP-ribose) polymerase-1 cleavage during apoptosis: An update. Apoptosis.

[28] Jungmichel S., Stucki M. (2010). MDC1: The art of keeping things in focus. Chromosoma.

[29] Solier S., Pommier Y. (2011). MDC1 cleavage by caspase-3: A novel mechanism for inactivating the DNA damage response during apoptosis. Cancer Res..

[30] Pines A., Vrouwe M.G., Marteijn J.A., Typas D., Luijsterburg M.S., Cansoy M., Hensbergen P., Deelder A., de Groot A., Matsumoto S. (2012). PARP1 promotes nucleotide excision repair through DDB2 stabilization and recruitment of ALC1. J. Cell. Biol..

[31] Reed E. (1998). Platinum-DNA adduct, nucleotide excision repair and platinum based anti-cancer chemotherapy. Cancer Treat. Rev..

[32] Van Waardenburg R.C., Meijer C., Burger H., Nooter K., De Vries E.G., Mulder N.H., De Jong S. (1997). Effects of an inducible anti-sense c-myc gene transfer in a drug-resistant human small-cell-lung-carcinoma cell line. Int. J. Cancer.

[33] Leonetti C., Biroccio A., Candiloro A., Citro G., Fornari C., Mottolese M., Del Bufalo D., Zupi G. (1999). Increase of cisplatin sensitivity by c-myc anti-sense oligodeoxynucleotides in a human metastatic melanoma inherently resistant to cisplatin. Clin. Cancer Res..

[34] Biroccio A., Benassi B., Amodei S., Gabellini C., Del Bufalo D., Zupi G. (2001). c-Myc down-regulation increases susceptibility to cisplatin through reactive oxygen species-mediated apoptosis in M14 human melanoma cells. Mol. Pharmacol..

[35] Reyes-González J.M., Armaiz-Peña G.N., Mangala L.S., Valiyeva F., Ivan C., Pradeep S., Echevarría-Vargas I.M., Rivera-Reyes A., Sood A.K., Vivas-Mejía P.E. (2015). Targeting c-MYC in platinum-resistant ovarian cancer. Mol. Cancer Ther..

[36] Yang X., Cai H., Liang Y., Chen L., Wang X., Si R., Qu K., Jiang Z., Ma B., Miao C. (2015). Inhibition of c-Myc by let-7b mimic reverses multidrug resistance in gastric cancer cells. Oncol. Rep..

[37] Kumari A., Folk W.P., Sakamuro D. (2017). The dual roles of MYC in genomic instability and cancer chemoresistance. Genes.

[38] Zanellato I., Colangelo D., Osella D. (2018). JQ1, a BET inhibitor, synergizes with cisplatin and induces apoptosis in highly chemoresistant malignant pleural mesothelioma cells. Curr. Cancer Drug Targets.

[39] Huang M.J., Cheng Y.C., Liu C.R., Lin S., Liu H.E. (2006). A small-molecule c-Myc inhibitor, 10058-F4, induces cell-cycle arrest, apoptosis, and myeloid differentiation of human acute myeloid leukemia. Exp. Hematol..

[40] Wang H., Hammoudeh D.I., Follis A.V., Reese B.E., Lazo J.S., Metallo S.J., Prochownik E.V. (2007). Improved low molecular weight Myc-Max inhibitors. Mol. Cancer Ther..

[41] Michels J., Vitale I., Galluzzi L., Adam J., Olaussen K.A., Kepp O., Senovilla L., Talhaoui I., Guegan J., Enot D.P. (2013). Cisplatin resistance associated with PARP hyperactivation. Cancer Res..

[42] Michels J., Vitale I., Senovilla L., Enot D.P., Garcia P., Lissa D., Olaussen K.A., Brenner C., Soria J.C., Castedo M. (2013). Synergistic interaction between cisplatin and PARP inhibitors in non-small cell lung cancer. Cell Cycle.

[43] Kalkat M., De Melo J., Hickman K.A., Lourenco C., Redel C., Resetca D., Tamachi A., Tu W.B., Penn L.Z. (2017). MYC deregulation in primary human cancers. Genes.

[44] Mota J.M., Barnett E., Nauseef J.T., Nguyen B., Stopsack K.H., Wibmer A., Flynn J.R., Heller G., Danila D.C., Rathkopf D. (2020). Platinum-based chemotherapy in metastatic prostate cancer with DNA repair gene alterations. JCO Precis. Oncol..

[45] Turner N., Tutt A., Ashworth A. (2004). Hallmarks of ‘BRCAness’ in sporadic cancers. Nat. Rev. Cancer.

[46] Helleday T. (2011). The underlying mechanism for the PARP and BRCA synthetic lethality: Clearing up the misunderstandings. Mol. Oncol..

[47] Lord C.J., Tutt A.N., Ashworth A. (2015). Synthetic lethality and cancer therapy: Lessons learned from the development of PARP inhibitors. Annu. Rev. Med..

[48] Larsen M.J., Thomassen M., Gerdes A.M., Kruse T.A. (2014). Hereditary breast cancer: Clinical, pathological and molecular characteristics. Breast Cancer.

[49] Ning J.F., Stanciu M., Humphrey M.R., Gorham J., Wakimoto H., Nishihara R., Lees J., Zou L., Martuza R.L., Wakimoto H. (2019). Myc targeted CDK18 promotes ATR and homologous recombination to mediate PARP inhibitor resistance in glioblastoma. Nat. Commun..

[50] Wang J., Ding Q., Fujimori H., Motegi A., Miki Y., Masutani M. (2016). Loss of CtIP disturbs homologous recombination repair and sensitizes breast cancer cells to PARP inhibitors. Oncotarget.

