# Supplementary material for: New Synthetic Lethality Re-Sensitizing Platinum-Refractory Cancer Cells to Cisplatin In Vitro: The Rationale to Co-Use PARP and ATM Inhibitors
Source: Int J Mol Sci. 2021 Dec 11;22(24):13324. doi: 10.3390/ijms222413324 (PMC8704450; doi:10.3390/ijms222413324)
Supplement: Supplementary file 1 [file ijms-22-13324-s001.zip › ijms-1439908-supplementary.pdf]

Supplementary Materials

New synthetic lethality re-sensitizing platinum-refractory cancer cells to cisplatin *in vitro*: the rationale to co-use PARP and ATM inhibitors

Watson P. Folk, Alpana Kumari, Tetsushi Iwasaki, Erica K. Cassimere, Slovénie Pyndiah, Elizabeth Martin, Kelly Homlar, Daitoku Sakamuro*

*Corresponding author

Figure S1: Ectopically expressed TP53 displays robust growth suppression in the presence of abundant BIN1 in DU145 prostate cancer cells.

Figure S2: Phase-contrast microscopy demonstrated that the cisplatin sensitivity obtained by si-MDC1 was counterbalanced by z-VAD-fmk (20 μM) in the LNCaP/sh-BIN1 cells.

Figure S3: Western blotting analysis probed with an anti-poly(ADP-ribosyl)ated (i.e., PARylated) carbohydrate monoclonal antibody (anti-PAR).

Figure S4: The mutation ratio of the *TP53*, *ATM*, *PARP1*, *MDC1*, *RNF8*, *BIN1*, and *MYC* genes in human solid cancer tissues.

Table S1: The chemicals used in this study

Table S2: The primary antibodies used in this study

Table S3: The si-RNAs/sh-RNAs used in this study

Supplementary Figures

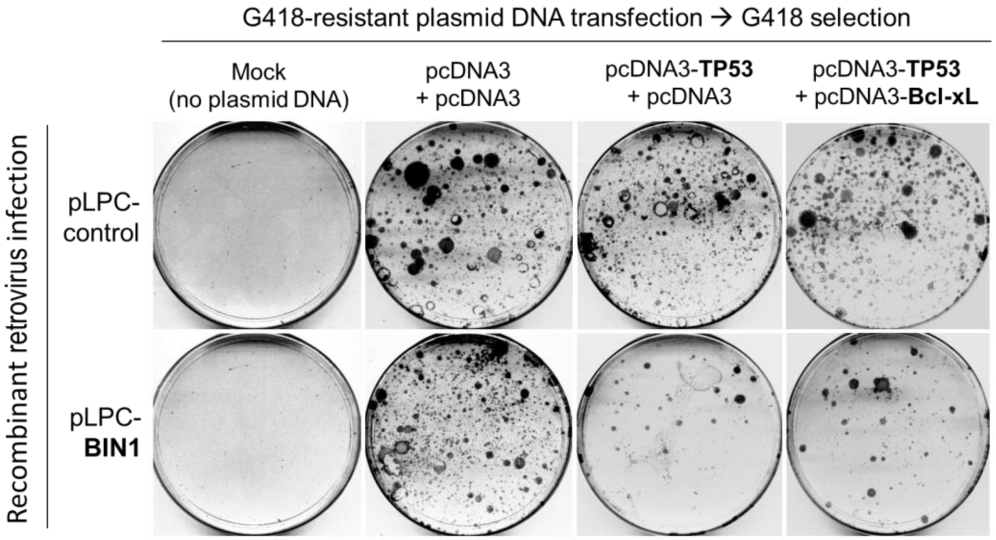

Figure S1 **Ectopically expressed TP53 displays robust growth suppression in the presence of abundant BIN1 in DU145 prostate cancer cells.** DU145 prostate cancer cells constitutively infected with the human full-length BIN1 cDNA-expressing recombinant retrovirus (pLPC-BIN1) were transfected with each indicated pcDNA3-based plasmid DNA vector (G418-resistant). Plasmid-transfected cells were incubated for 2-3 weeks in the presence of G418 (500 μg/mL) under optimal culture conditions. G418-resistant colonies that appeared on tissue culture plates were fixed briefly with 100% methanol and then stained with Giemsa (Invitrogen, Carlsbad, California, USA) according to the protocol recommended by the vendor. Besides rapid induction of G1 cell-cycle arrest when overexpressed or in response to DNA damage, the TP53 tumor suppressor (also known as p53) activates apoptosis that would be counteracted by Bcl-xL protein [S1]. Bcl-xL (B-cell lymphoma-extra-large) protein acts as an anti-apoptotic protein by blocking the release of cytochrome c from mitochondria [S2].

[S1] Vaseva AV, Moll UM (2009) The mitochondrial p53 pathway. *Biochim Biophys Acta* 1787: 414-420. https://doi.org/10.1016/j.bbabio.2008.10.005

[S2] Korsmeyer SJ. (1995) Regulators of Cell Death. *Trends in Genetics* 11: 101-105. https://doi.org/10.1016/S0168-9525(00)89010-1

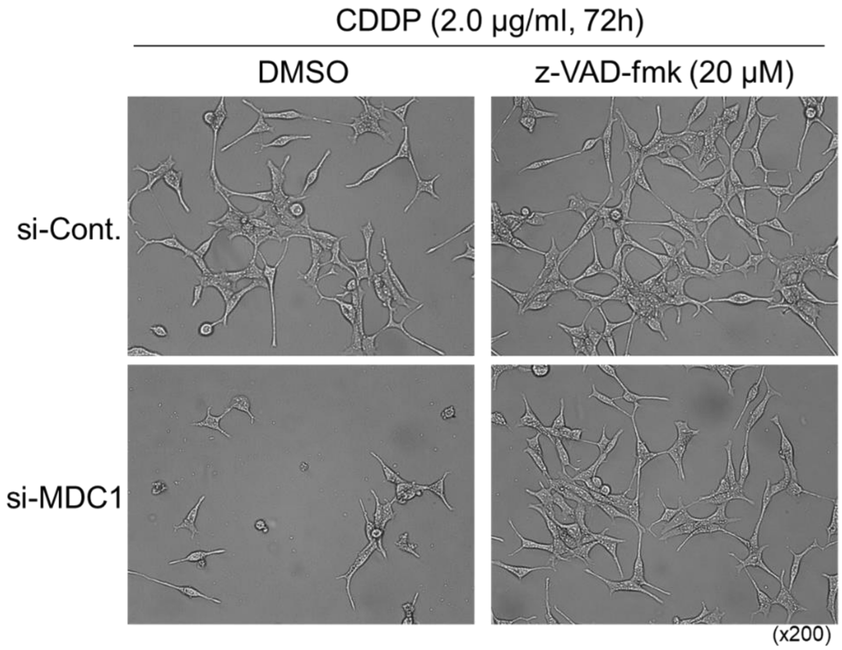

Figure S2 **Phase-contrast microscopy demonstrated that the cisplatin sensitivity obtained by si-MDC1 was counterbalanced by z-VAD-fmk (20 µM) in the LNCaP/sh-BIN1 cells.** This morphological observation indicates that MDC1 depletion elicits cisplatin-induced apoptosis in a caspase-dependent manner, thereby counteracting the cisplatin resistance by a BIN1 loss.

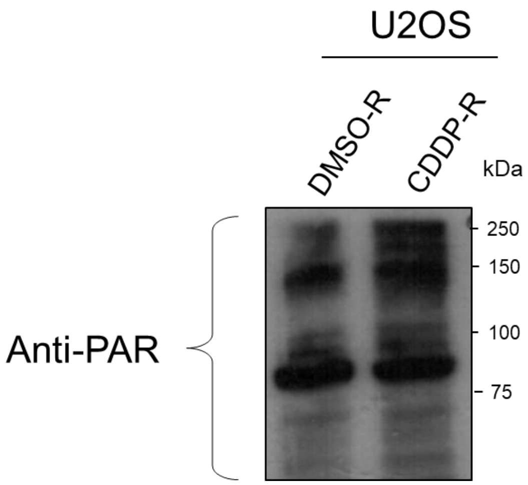

Figure S3 **Western blotting analysis probed with an anti-poly(ADP-ribosyl)ated (i.e., PARylated) carbohydrate monoclonal antibody (anti-PAR).** This observation indicates that endogenous PARP1 activity increases during the process for cancer-cell survival in the presence of cisplatin.

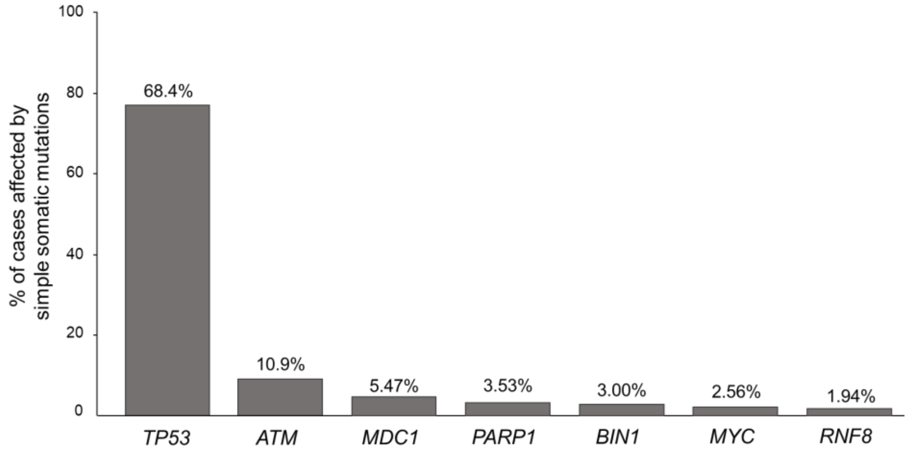

Figure S4. **The mutation ratio of the *TP53*, *ATM*, *PARP1*, *MDC1*, *RNF8*, *BIN1*, and *MYC* genes in human solid cancer tissues.** The updated data of the gene mutation ratios in human cancer tissues derived from various organs, such as lung, breast, bone marrow, colon, nervous system, ovary, kidney, skin, and prostate, are available at the US National Cancer Institute (NCI) Genomic DNA Commons (https://gdc.cancer.gov/). Simple somatic gene abnormality includes missense mutations, frameshift mutations, start-loss mutations, stop-loss mutations, and stop-gained mutations.

Supplementary Tables

Table S1: The chemicals used in this study

Chemical	Catalog number	Company
Dimethyl sulfoxide (DMSO)	sc-358801	Santa Cruz Biotechnology
Olaparib (PARP inhibitor)	sc-302017	Santa Cruz Biotechnology
4’-6-diamino-2-phenylindole (DAPI)	D8417	Sigma-Aldrich
G418 (Geneticin)	#10131027	Thermo Fisher Scientific
Cisplatin	P4394	Sigma-Aldrich
Bleomycin	B-2434	Sigma-Aldrich
Z-VAD-fmk	V-116	Sigma-Aldrich
KU-60019 (ATM inhibitor)	#4176	Tocris Bioscience

Table S2: The primary antibodies used in this study

Antigen detected	Catalog number	Company
BIN1 (clone 99D)	sc-13575	Santa Cruz Biotechnology
BIN1 (clone D3)	sc-74486	Santa Cruz Biotechnology
PARP1	sc-7150	Santa Cruz Biotechnology
PAR	4335-MC-100	Trevigen
γH2AX	#2577	Cell Signaling Technology
ATM	05-513	Millipore
MDC1	ab114143	Abcam
Phospho-MDC1	ab35967	Abcam
RNF8	sc-271462	Santa Cruz Biotechnology
MYC (9E10)	MA1-980	Thermo Fisher Scientific
TP53 (DO-1)	sc-126	Santa Cruz Biotechnology
Phospho-TP53 (Ser15)	#9284	Cell Signaling Technology
ERCC1 (D-10)	sc-17809	Santa Cruz Biotechnology
GAPDH	sc-322233	Santa Cruz Biotechnology
β-Actin	A2228	Sigma-Aldrich

Table S3: The si-RNAs/sh-RNAs used in this study

sh-/si-RNA	Catalog number	Company
BIN1 sh-RNA (human)	sc-29804-SH	Santa Cruz Biotechnology
MDC1 si-RNA (human)	sc-43917	Santa Cruz Biotechnology
RNF8 si-RNA (human)	sc-61484	Santa Cruz Biotechnology
